# Staged and geometry-adapted support for coal mine roadways under weak rock conditions: a numerical study

**DOI:** 10.1038/s41598-025-30587-0

**Published:** 2025-12-22

**Authors:** Yu Guo, Qinghua Ma, Shuai Zhong

**Affiliations:** 1 College of Architecture and Construction, Jiangsu Vocational Institute of Architectural Technology, Xuzhou, 221116 P. R. China; 2 College of Safety Engineering, Lanzhou Resources & Environment Voc-tech University, Lanzhou, 730123 P. R. China

**Keywords:** Coal mine roadway, Staged support strategy, Weak rock mass, Numerical simulation, Deformation control, Stress redistribution, Energy science and technology, Engineering, Solid Earth sciences

## Abstract

Controlling large deformation and instability of roadways in weak coal seams remains a critical challenge for safe and efficient mining operations. This study carried out an engineering filed observation firstly, and by setting three main roadways as research targets, it then proposed a series of supporting strategies for each of them. Numerical model was established using RockScience Phase2 finite element software to simulate the roadway’s mechanical response across all stages. Results indicated that the staged supporting strategy sequentially mitigated excavation-induced instability, roadway widening increased rib convergence, roadway heightening triggered additional roof sag. Supplementary grouting and rib bolts were testified as effective. Stress redistribution analysis revealed that grouting and bolts transferred concentrated stress from the weak coal seam to the stiff sandy mudstone. Additionally, a comparative analysis of a trapezoidal roadway variant validated the universality of the hybrid ‘liner + grouting’ support, which reduced yielded elements compared to unsupported conditions. This study demonstrates that phased support, tailored to excavation sequence and geological weakness, is an effective solution for roadway stability control, providing theoretical and technical references for similar weak rock mining projects.

## Introduction

Underground coal mining roadways serve as critical infrastructure for material transportation, ventilation, and personnel access, and their long-term stability directly determines mining efficiency and operational safety^1–3^, especially in weak rock mass environments^4^. Weak rock formations (e.g., low-strength coal seams and fractured sandy mudstone) are characterized by high porosity, low compressive/tensile strength, and strong time-dependent deformation^5–7^. When subjected to excavation-induced stress redistribution and recurrent front abutment pressure from retreat mining, these formations often exhibit severe failure phenomena^8–10^, such as roof delamination, rib spalling, floor heave, and cross-sectional shrinkage. For roadway support under such conditions, a more scientific support design is required. This encompasses stress environment assessment prior to coal mine development, coordinated control of the mining sequence to optimize mining-induced stress^11^, and a rational step-by-step design of the support installation sequence. Each stage plays a crucial role in ensuring the eventual stability of the roadway.

In the Daxi Coal Mine (Shanxi Province, China), for instance, the main haulage, ventilation, and conveyor roadways, which were all surrounded by a 5 m-thick coal seam and underlain and overlain by sandy mudstone, have experienced persistent instability, as initial support schemes failed to prevent progressive deformation. A core challenge in current engineering practice lies in the lack of tailored support design: most schemes adopt fixed configurations (e.g., single rock bolts) without accounting for dynamic excavation processes (e.g., sequential widening and heightening) or geometric variations (e.g., rectangular vs. trapezoidal cross-section)^12^. This mismatch between support and actual engineering conditions leads to repeated remediation, increased costs, and potential safety hazards, highlighting the urgency of developing adaptive stability control strategies for weak rock roadways^13^.

In recent years, significant progress has been made in understanding and mitigating weak rock roadway instability^14–16^. Numerically, finite element and finite difference software have become indispensable tools for simulating the mechanical behavior of surrounding rock^17–19^, enabling researchers to quantify stress^20–22^, deformation^23^, and failure evolution under different support configurations^24–26^. These existing research have been widely used to evaluate the efficacy of common support measures, such as rock bolts (for localized mechanical constraint)^27^, cable bolts (for deep load transfer)^28^, and grouting (for improving rock mass integrity). Studies have shown that grouting can effectively fill fractures in weak rock^29^, enhance its cohesion and internal friction angle, and redistribute concentrated stress away from the excavation boundary^30^. Meanwhile, hybrid support systems combining rigid liners with grouting or bolts with cables have been proven more effective than single-component schemes in controlling large deformation^31^–^32^, as they integrate passive stiffening (liner/grouting) and active restraint (bolts/cables)^30^. Additionally, research on staged support has emphasized the importance of synchronizing support intervention with excavation sequence, arguing that dynamic adjustments to support intensity can better address the time-dependent and cumulative instability of weak rock^33^. However, most existing studies focus on idealized geological conditions or single excavation scenarios, with limited attention to the coupling effects of progressive excavation (e.g., widening followed by heightening) and geometric variations (e.g., trapezoidal sections for highly fractured rock), which are common in practical mining engineering.

Despite advances in soft rock roadway support, two key limitations persist: first, current research rarely links support design to the actual dynamic excavation process of roadways^34^, leading to schemes that cannot adapt to cumulative stress and deformation; Second, there is a lack of systematic validation for support schemes tailored to non-rectangular cross-sections (e.g., trapezoidal roadways in highly fractured zones). To address these gaps, this study takes the Daxi Coal Mine as a case study, using RockScience Phase2^35^ to simulate a six-stage support strategy for rectangular roadways and a hybrid ‘liner + grouting’ scheme for trapezoidal roadways. The innovation lies in integrating field observation data with numerical simulation to develop adaptive support schemes that match excavation sequence and geometric characteristics, providing a practical reference for weak rock roadway stability control.

## Engineering background

**Fig. 1:**
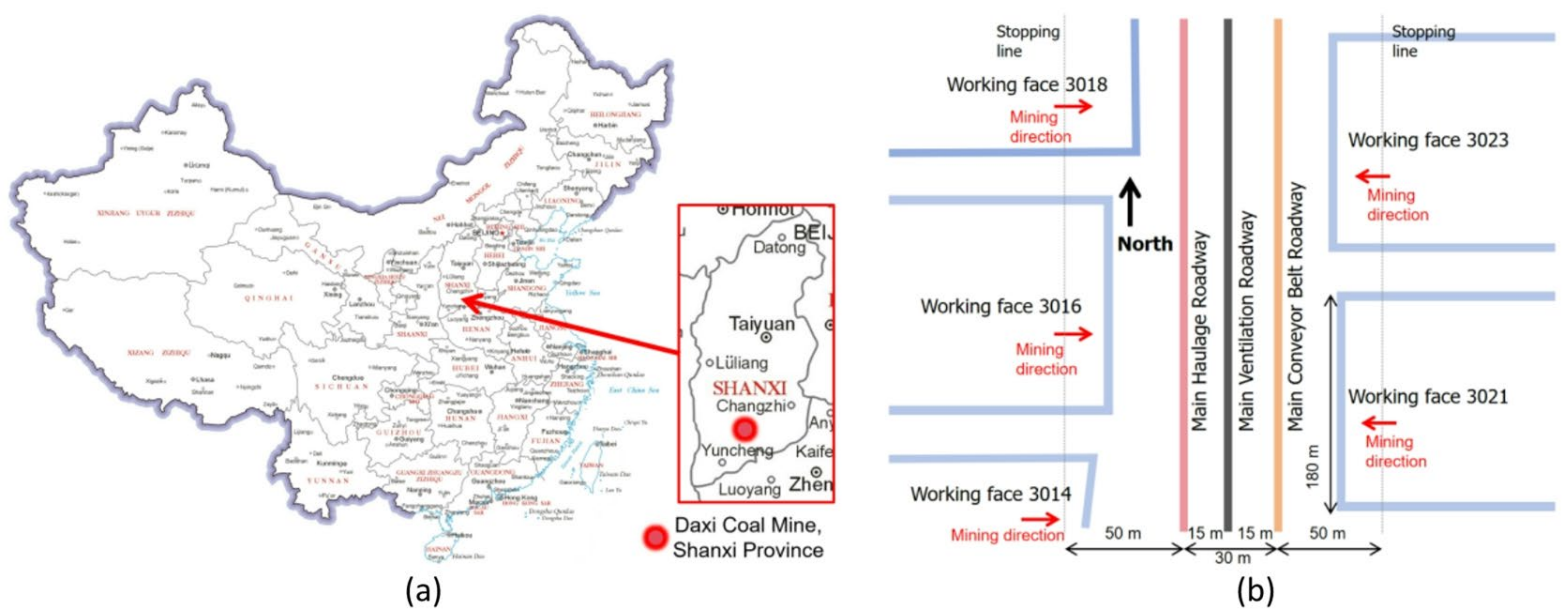
Geological location of the Daxi Coal Mine on the map of China (**a**) and the engineering layout of roadways (**b**).

Daxi Coal Mine is situated on the northwestern side of Yangcheng County, Shanxi Province, as marked in Fig. 1a, The source of the map is accessible via the link http://bzdt.ch.mnr.gov.cn. The mine has an annual coal production capacity of 600,000 tons. The coal seam has an average burial depth of 300 m, an average ground elevation of 600 m, an average thickness of 5 m, and a dip angle of 5°. The immediate roof and floor of the coal seam are both sandy mudstone, with average compressive strengths of 28 MPa and 24 MPa, respectively.

The research was conducted in three main roadways, namely the main haulage roadway, main ventilation roadway, and main conveyor belt roadway (see Fig. 1b). All mining faces adopt the retreat mining method, which induces significant stress disturbance and thus affects the stability of these three main roadways. Meanwhile, these three roadways serve as the key infrastructure of the entire panel, meaning their designed service life should be consistent with that of the panel. Field in-situ stress monitoring results show that the vertical stress is 7.32 MPa, the maximum and minimum horizontal principal stresses are 9.28 MPa and 5.32 MPa, respectively, and the maximum horizontal principal stress acts in the direction of N35.8°E.

**Fig. 2:**
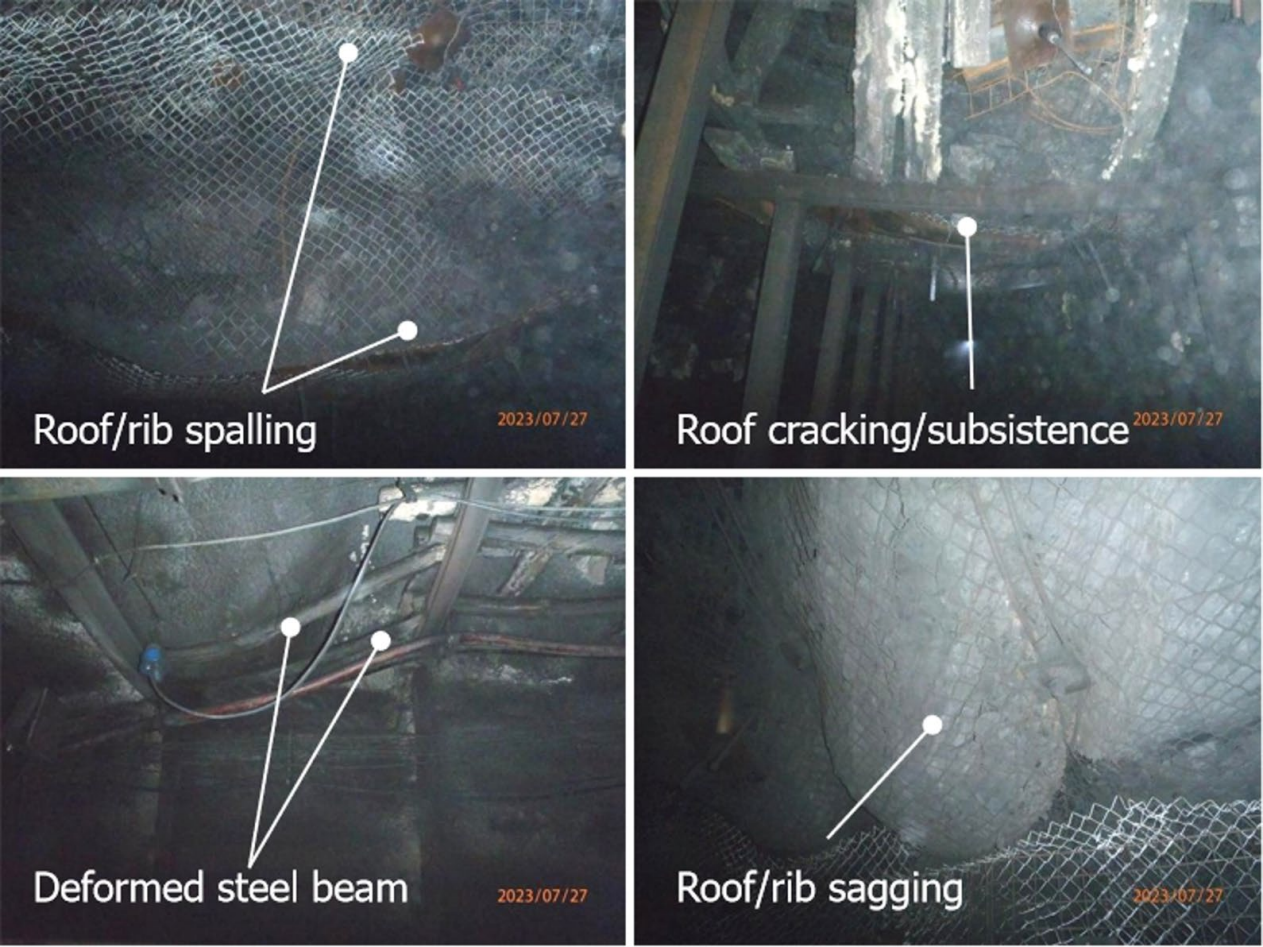
Failure patterns of roadways in engineering site.

Affected by the severe front abutment pressure from the mining faces (see Fig. 1b), the roadways were subjected to long-lasting and recurrent stress disturbances. This ultimately led to prominent roadway failure, as shown in Fig. 2. Specific failure phenomena included: roof delamination and spalling in some sections; insufficient roof support capacity in others, which induced roof cracking and subsidence; deformation of steel beams in partial areas; and more critically, widespread roof and rib sagging across the roadways—an issue attributed to inadequate initial active support.

**Fig. 3:**
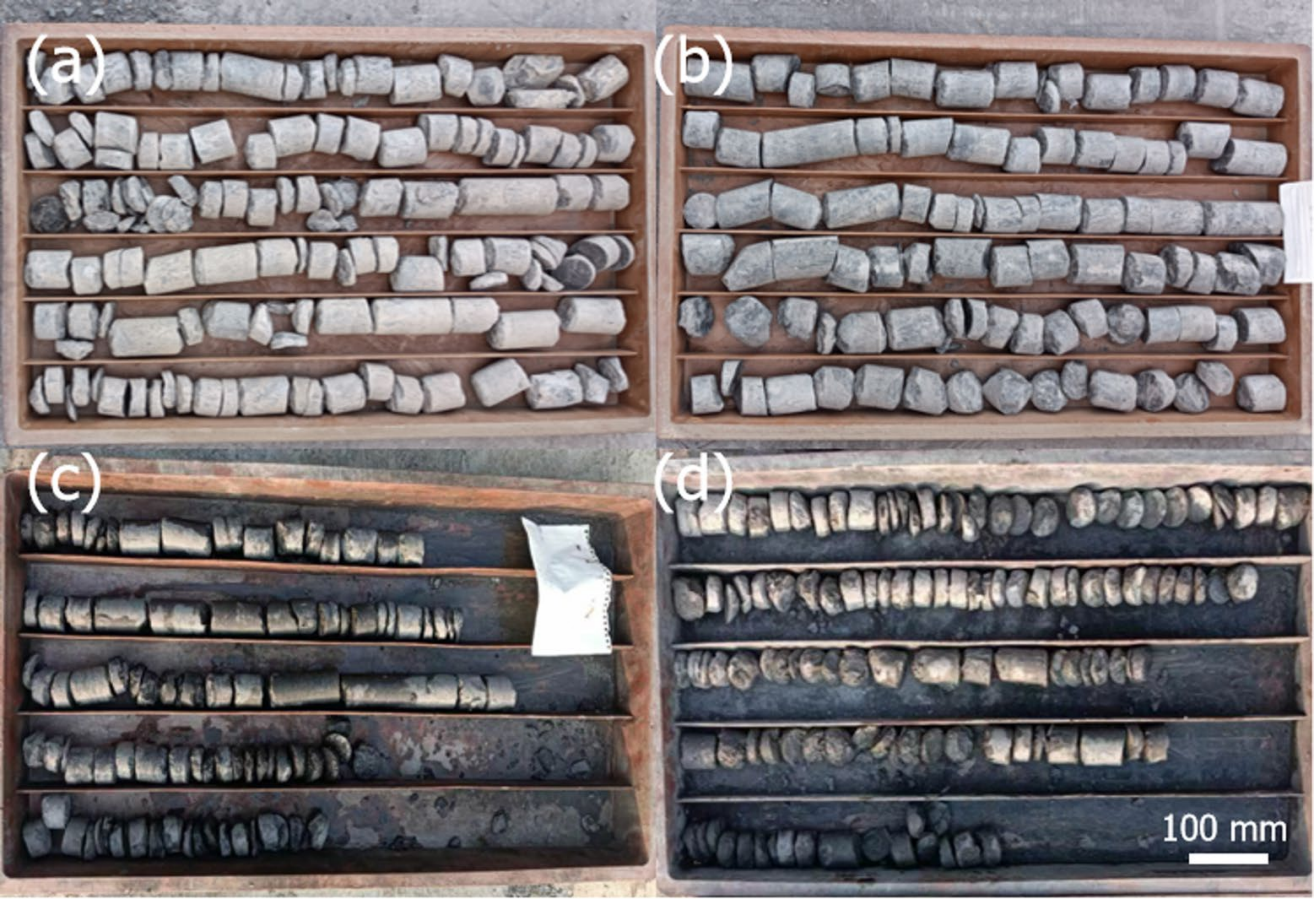
Exhibition of drilled cores, (**a**-**d**) rock disks from boreholes No.1-No.4, respectively.

To further clarify the mechanism underlying roadway failure, drilling tests were conducted in selected sections of the roadways. The drilling depth ranged from 15 m to 30 m, with a fixed drill bit diameter of 56 mm. Drilling depth variations (15–30 m) was resulted from roof strata heterogeneity. Shorter cores and darker color were due to mudstone in these sections, mudstone’s weak cementation also caused incomplete core recovery, while its inherent lithology led to the darker appearance.A total of four boreholes were completed: the 1 st and 4th boreholes were located in the main conveyor belt roadway (Fig. 1b), while the 2nd and 3rd were in the main haulage roadway (Fig. 1b). As presented in Fig. 3, the drilled cores reveal that the roof strata were highly fractured and exhibited low rock strength. This poor rock quality made it difficult to obtain intact cored rock disks longer than 100 mm.

**Fig. 4:**
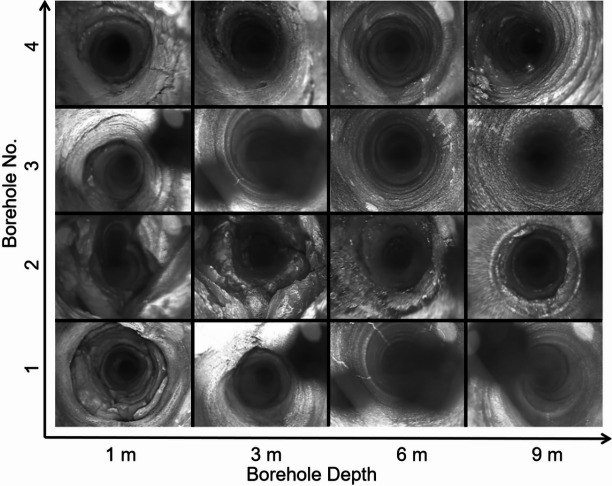
Borehole imaging photos vs. borehole depth.

Figure 4 presents the borehole imaging data of the four boreholes (Nos. 1–4), where Nos. 1–2 were drilled in the main haulage roadway and Nos. 3–4 in the main conveyor roadway. The distances of these boreholes from the starting point of their respective roadways were 1350 m, 1550 m, 2100 m, and 2150 m, respectively. The starting point (0 m) here indicated the very beginning of each roadway. Each borehole had a diameter of 50 mm and a length of 10 m. The imaging results indicate a high degree of fracturing in all boreholes, particularly within the depth range of 0–3 m. Even at a depth of 9 m, significant fracturing persisted in boreholes Nos. 1, 2, and 4.

In light of the identified roadway failure patterns and the internal fracturing state of the surrounding rock mass, the existing support scheme must be upgraded to address these challenges. In the subsequent sections, numerical simulation tools will be employed to explore the optimal support measures for different failure scenarios.

## Analytical methods and results

### Numerical model construction of staged support strategy (main haulage roadway)

This section utilizes the finite element software Phase2 (RockScience) to conduct a numerical investigation into the stability of the main haulage roadway under six staged intervention measures, as elaborated in subsequent sections. RockScience’s Phase2 is a finite element software for geomechanical analysis, specializing in assessing the stability of underground excavations like mine roadways. It models stress redistribution, rock deformation, and failure, while integrating support systems (bolts, grouting) to optimize stability strategies, making it vital for mining and tunneling projects. In this study, Phase2 enabled seamless integration of multi-stage excavation and support processes, as well as accurate simulation of stress-deformation behavior in weak rock masses. Compared with FLAC/UDEC for this specific weak rock roadway scenario, Phase 2 was all more straightforward.

**Fig. 5:**
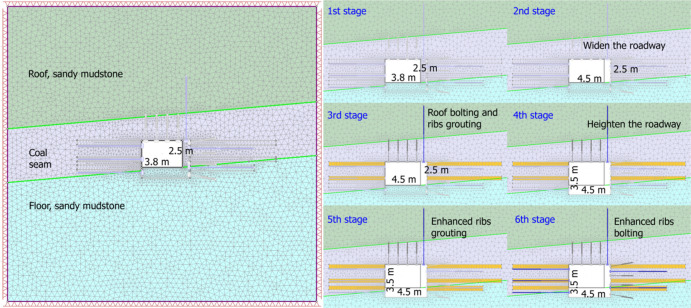
Numerical model of staged support strategy for a failed roadway.

Based on the engineering prototype, a geological setting was constructed that comprises a roof of sandy mudstone, a coal seam, and a floor of sandy mudstone, as illustrated in Fig. 5 (left subfigure). The protocol is outlined here; initially, the designed width and height were set at 4.5 m and 3.5 m respectively. However, due to continuous deformation, it became necessary to re-excavate the roadway to restore its original design geometry, necessitating an upgrade in the supporting pattern. Consequently, for this analysis, six distinct stages are delineated in Fig. 5 (right subfigure): (1) initial excavation (3.8 m wide, 2.5 m high); (2) widening the roadway to 4.5 m; (3) installing roof bolts and grouting ribs; (4) heightening the roadway to 3.5 m; (5) enhancing ribs with additional grouting; and (6) reinforcing ribs with rock bolts (gray) and cable bolts (blue). The roof bolting in stage 3 is set for the concern of deterioration because of widening the right rib (Stage 2). Roof bolting is the fastest and most constructible measure to avoid severe roof deformation. In addition, mesh support in Fig. 2 was not incorporated into numerical model, because its primarily function was to prevent minor rock spalling, it was a local, non-structural effect, and the exclusion does not compromise simulation accuracy.

It is important to note that the blue-colored long bolt in the third stage is a cable bolt with a length of 9 m and a diameter of 21.8 mm. The grouting process in the simulation was achieved by assigning relatively higher strength parameters to the materials represented by the yellow-colored bar. This grouting simulation simplification is reasonable because grouting in weak coal seams primarily fills fractures and enhances rock mass cohesion and internal friction angle, whose macroscopic effect is equivalent to improving strength parameters—a common practice in mining geomechanical simulations. Compared with advanced models such as FLAC/UDEC, this method avoids complex seepage-stress coupling, facilitates multi-stage support simulation, and focuses on macro stability rather than micro mechanisms, thereby capturing the key mechanical role of grouting.

In practical engineering applications, grouting was performed using cables measuring 6 m in length and having a diameter of 42 mm. Considering a conservative expansion zone for the grouting material that is approximate five times its original size, the yellow-colored bar had dimensions of 210 mm in width and 6 m in length eventually. For rock bolts, the diameter was specified as 22 mm and their length as 2200 mm. In the sixth stage, cable bolts measured 4.5 m in length with a diameter of 21.8 mm. All bolts were bonded using resin cartridges, and they were all prestressed after installation.

By examining deformation, stress, strength factor, and support performance at each stage, the efficacy of this phased support strategy in controlling excavation-induced instability was quantified.

### Numerical evaluation of original supporting pattern

**Fig. 6:**
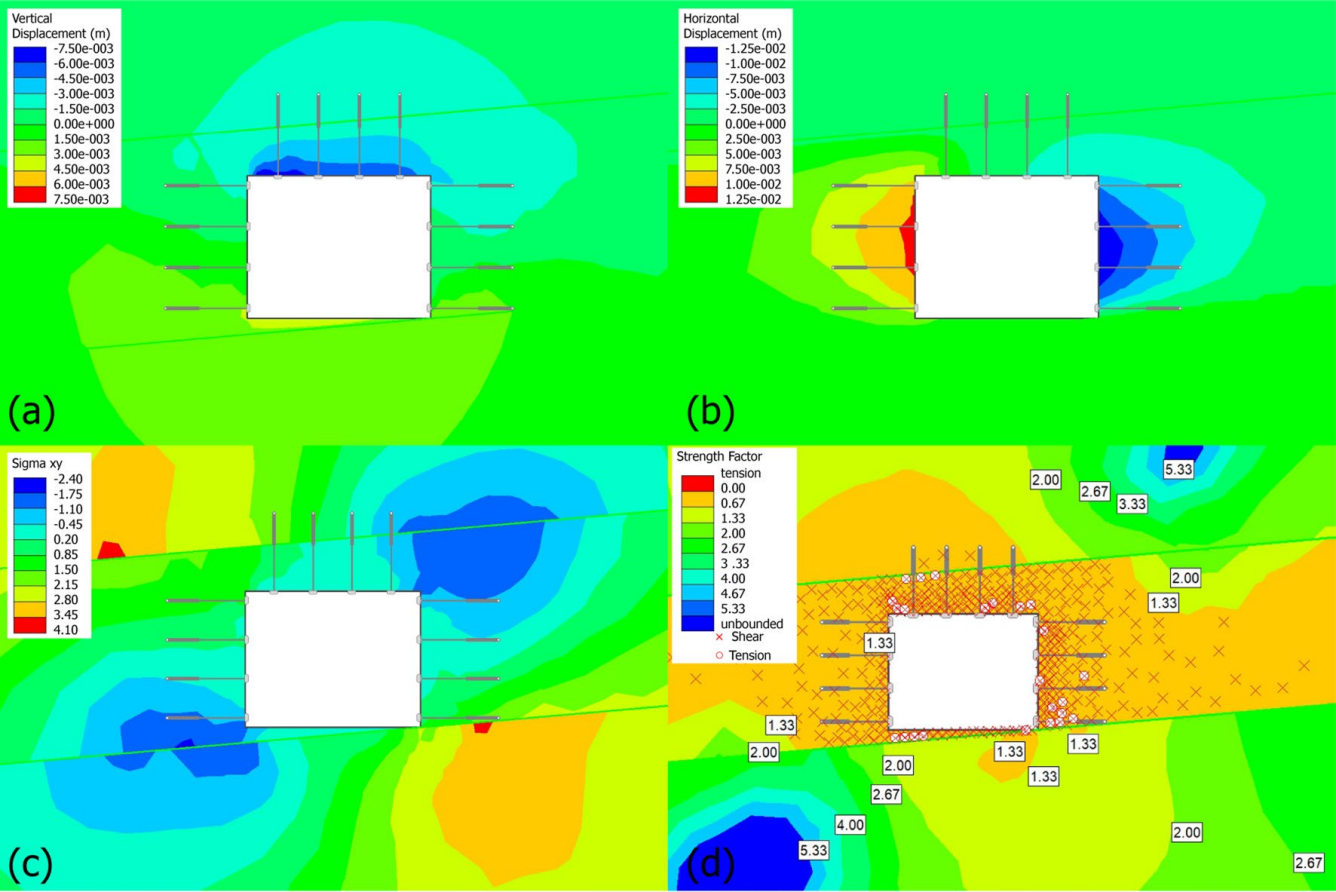
Numerical contour maps showing different evaluating factors, (**a**) vertical displacement, (**b**) horizontal displacement, (**c**) shear stress, (**d**) strength factor with yielded elements.

In the next section, the numerical model of the staged support strategy for failed roadways would be simulated. However, the cause of roadway failure must be investigated prior to this simulation. The original roadway was supported solely by rock bolts, with a designed width of 4.5 m and a designed height of 3.5 m. Specifically regarding the support configuration, 12 rock bolts were installed on each of the roof, left rib, and right rib, with 4 bolts arranged on each side at an interval of 1 m. All bolts had a length of 2200 mm and a diameter of 22 mm, and their ends were encapsulated using resin cartridges. The simulated results are exhibited in Fig. 6.

For the vertical displacement results (Fig. 6a), maximum vertical displacement is concentrated in the roof (dark blue contours), indicating significant downward movement due to excavation. The floor shows smaller displacement, reflecting limited deformation. In addition, rock bolts effectively reduce displacement in the roof by redistributing stresses and preventing excessive deformation, as evidenced by gradient changes near the bolts. Overall, the roof experiences significant deformation but remains stabilized by the bolts. The floor shows minimal movement, suggesting sufficient rock mass strength below the excavation.

For the horizontal displacement results presented in Fig. 6b, significant horizontal displacements occur on the ribs of the coal seam, with maximum values shown in red and blue contours on either side. This indicates lateral movement toward the excavation’s interior. Displacement in the roof and floor is minimal (green contours), reflecting their relatively higher strength compared to the coal seam. Rock bolts installed in the roof do not directly influence horizontal displacement in the sidewalls, therefore the horizontal shearing tendency in the roof should be minimal. The deformation is concentrated around the coal seam ribs, with displacement rapidly diminishing in the stronger roof and floor, hence, these layers act as natural confinements.

Figure 6c shows the shear stress distribution. As can be seen, high magnitudes of shear stress are concentrated near the sidewalls of the excavation, as indicated by the dark blue and orange contours. The roof and floor exhibit minimal shear stress (green regions), consistent with their higher strength and confinement effect on the weaker coal seam. The contribution of the rock bolts is most evident in the roof, where they prevent the development of high shear stresses by stabilizing the roof region. However, the bolts have limited direct influence on the ribs, where significant shear stresses persist. The ribs of the coal seam are the primary zones of concern, as they experience the highest shear stresses. This is due to the lower strength of the coal seam and the stress concentration caused by excavation-induced deformation. High shear stresses in the ribs suggest a potential for shear failure in these regions, particularly in the weaker coal seam. Reinforcement measures (e.g., additional bolts or shotcrete) should target these areas to mitigate failure.

Figure 6d gives the strength factor distribution. The strength factor is lowest around the sidewalls and the corners of the excavation (values near 1.0). These areas are most critical, as they are closest to failure under the current stress state. The roof and floor show higher strength factor values (greater than 2.0) due to their greater inherent strength compared to the coal seam. Areas with a strength factor near 1.0 are likely experiencing shear failure, as indicated by the crosshatched zones in red. These zones are concentrated in the weaker coal seam around the excavation boundaries.

Therefore, the bolts do not significantly reinforce the weaker ribs, where the strength factor remains low. The roof and floor experience no apparent failure in this simulation, however, it should be mentioned that one most important criterion of controlling roof is to control the ribs, and ribs long-last deformation has decisive impacts on roof’s sagging, which then leads to overall failure of whole roadway. In high-stress or weak rock conditions, supplementary support (e.g., cable bolts or shotcrete) should be considered. In this case, Additional support (e.g., denser bolt spacing, shotcrete, or mesh) is required for the ribs to prevent failure in these critical zones. As for the roof, the bolts are effective, but additional reinforcement (e.g., longer bonded lengths or denser bolt spacing) may be required to further minimize roof displacement.

### Numerical evaluation of staged support strategy in main haulage roadway

**Fig. 7:**
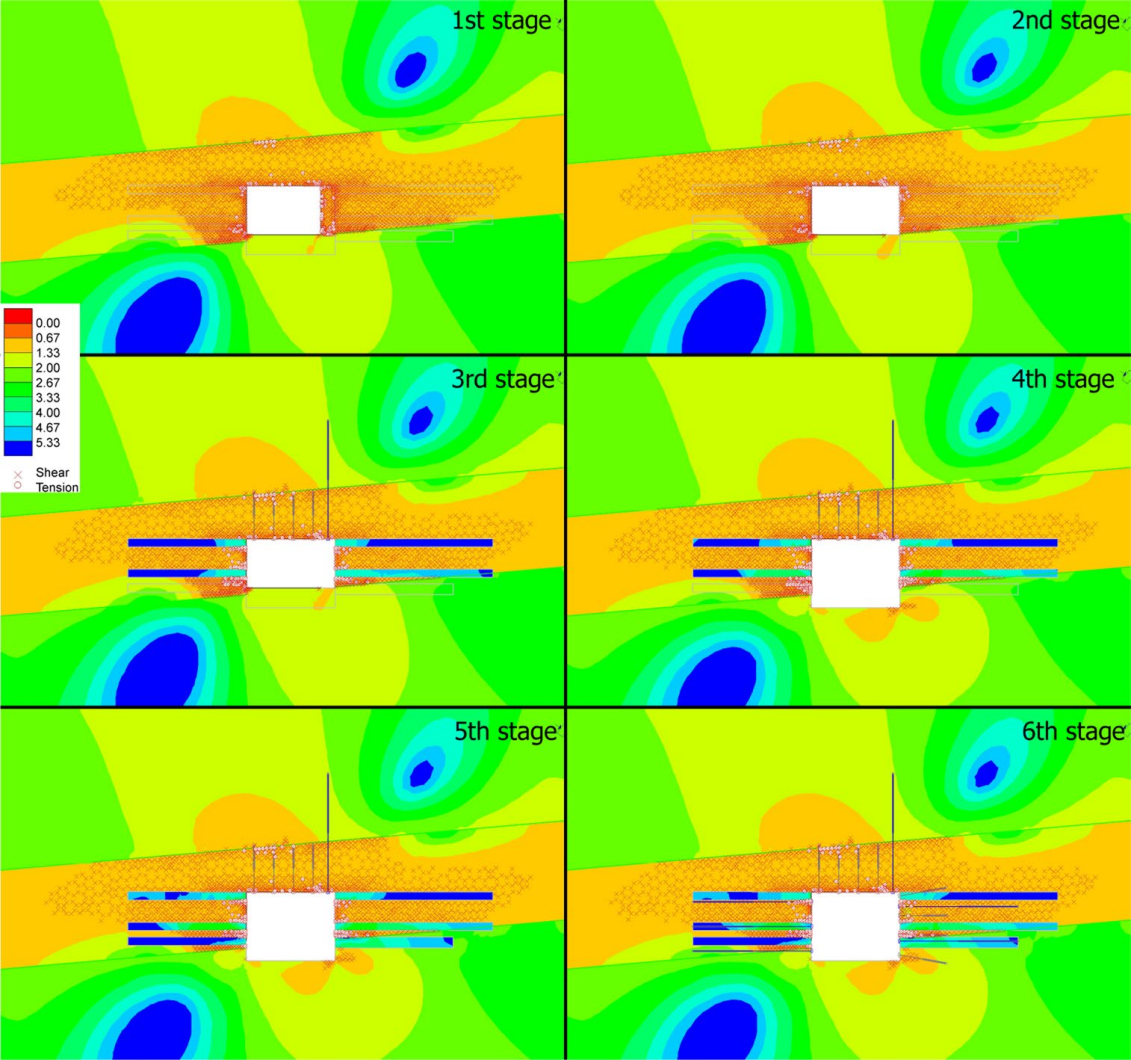
Contour maps of numerical simulation results showing strength factor variations at each stage.

As discussed above, the original support scheme is unable to meet the stability control requirements of the main haulage roadway, it was a conclusion further validated by the engineering observations presented in Fig. 2. Specifically, the roadway exhibited severe deformation: its cross-sectional dimensions shrank from the designed 4.5 m × 3.5 m to 3.8 m × 2.5 m (see Fig. 5), necessitating a remediation procedure. Against this background, the staged support strategy proposed in Fig. 5 was adopted, and the numerical simulation results of the strength factor (SF) variations and yielded elements across different stages are provided in Fig. 7.

#### Strength factor

For initial failed roadway geometry (3.8 m × 2.5 m in stage 1), SF distribution indicates that the coal seam exhibits widespread orange (SF ≈ 0.67–1.33) and red (SF < 0.67) zones, indicating marginal-to-failure stability. The roof/floor sandy mudstone shows higher SF (green-to-blue), confirming coal as the weak link. In terms of the yielded elements, dense shear (crosses) and tension (circles) yield zones at coal-rib corners, driven by stress concentration in the low-strength coal under field stress. Afterwards, the width of the roadway is widened to 4.5 m (right rib, stage 2), the right coal rib shows expanded orange/red SF zones, as widening amplifies horizontal stress concentration. The left rib remains relatively stable, highlighting asymmetric stability loss from span increase^36^. For the yielded elements, Yield zones extend further at the right rib, it thus confirms that span increase exacerbates coal failure. The roof/floor remain stable in this stage.

In stage 3, rib grouting and roof supports are installed, grouting introduces blue/green SF zones at ribs, transferring load from coal to grout. Roof supports redistribute roof stress, reducing SF gradients. Yield zones are drastically reduced, especially at the right rib. Grouting stiffens the coal-rib interface, while roof supports control roof sag, the efficacy of combined grouting and roof reinforcement are demonstrated. In stage 4, floor excavation is carried out to increase the height to 3.5 m. Taller excavation introduces new orange/red SF zones at lower coal ribs and the new floor. Vertical stress amplification (from taller overburden) challenges the coal seam’s low strength, driving SF below 1 in these regions. New yield zones appear at lower ribs, as combined vertical and horizontal stress exceeds coal’s capacity. The floor sandy mudstone remains stable, but coal failure propagates downward.

Lower rib grouting is added in stage 5, grouting the lower ribs mirrors stage 3, blue/green SF zones emerge at lower grouted regions, restoring S ≥ 1. Symmetry in grouted reinforcement (upper and lower ribs) improves overall stability. Lower rib yield zones are confined, because the grout transfers stress from coal to the stiffer grout matrix. This replicates the success of upper-rib grouting, the stability of the taller roadway’s lower regions is guaranteed. By adding rib bolts and cable bolts in the stage 6, rib bolts tensile capacity and cable bolts introduce uniform high-SF zones (blue/green) along all ribs. Active tensile resistance from supports restrains convergence, eliminating SF < 1 zones. Because bolts and cables provide direct load resistance, the yield zones are minimized. This supporting system achieves full stability, with no localized failure in coal or grout.

From above observations, it can be seen that grouting effectively transfers load from coal to grout, reducing yield and restoring SF. Its impact is consistent across upper and lower ribs. Secondly, the roof/cable bolts and rib bolts work synergistically, roof supports control sag, while rib supports restrain convergence, creating an effective stress-redistribution network. It also demonstrates that by re-shaping the roadway, the widening and heightening excavations increase stress concentration in coal, but targeted reinforcement (grouting + supports) mitigates these effects, therefore the importance of stage-wise intervention in weak-rock excavations is emphasized.

#### Absolute horizontal displacement

**Fig. 8:**
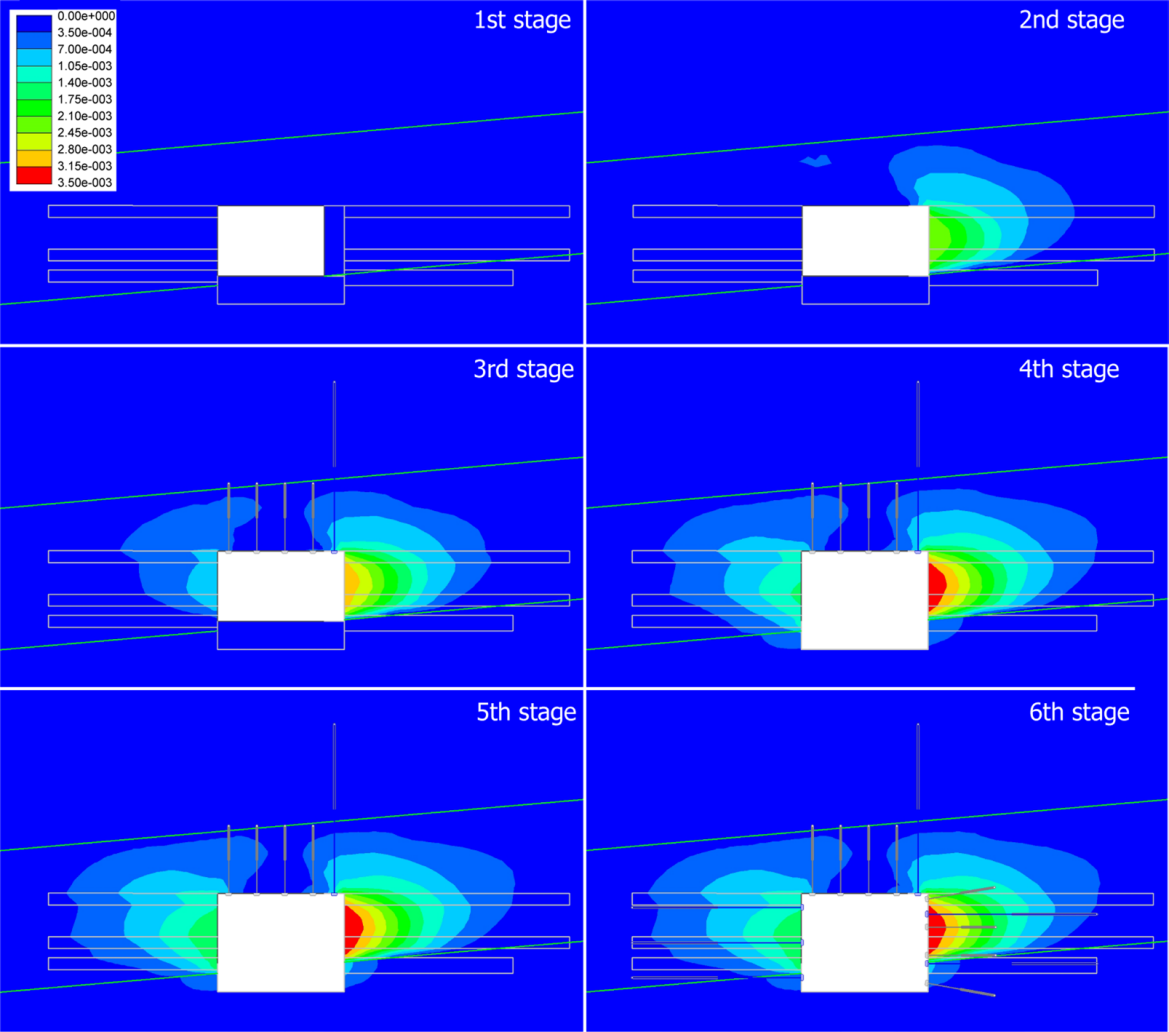
Contour maps of numerical simulation results showing absolute horizontal displacement variations at each stage (unit in meters).

Figure 8 exhibits the absolute horizontal displacement variations at each stage. The first stage is set as referred stage indicating displacement of 0. For the stage 2 (widening to 4.5 m of right rib), asymmetric gradient (blue-green-yellow) is noticed at the right rib. The left rib remains blue. Widening the span amplifies horizontal stress concentration at the right rib. The coal seam’s low strength make it susceptible to convergence. For the stage 3 (rib grouting + roof supports), the displacement gradient at the right rib is reduced (fewer yellow zones). The left side now shows blue-to-cyan displacement. Grouting stiffens the coal-rib interface, transferring load from coal to grout. Roof supports redistribute roof stress, limiting indirect rib deformation. For the stage 4 (floor excavation to a height of 3.5 m), a red zone (3.5 mm) emerges at the right rib, with displacement extending to lower regions. Increased height amplifies vertical stress, and together with horizontal stress, which leads to increased overburden height that drives more pronounced rib convergence. In stage 5 (lower rib grouting), the red zone at the right rib is marginally reduced, and displacement contours are confined. The grouting restricts deformation propagation downward, extending stability to the taller excavation. In stage 6 (rib bolts + cable bolts installation), the displacement intensity drops significantly (red - green/cyan), with both ribs showing minimal deformation. The supports provide active tensile resistance, restraining rib convergence. This synergizes with grouting (passive stiffness) to achieve near-elastic deformation^37^, and it indicates that the combined mechanical and grouted reinforcement are efficient.

In summary, approaches such as widening (Stage 2) and heightening (Stage 4) roadways exacerbate horizontal displacement, with the widened side and lower ribs being most vulnerable. For grouting, it effectively transfers load from weak coal to stiff grout, the displacement has been reduced by more than 50% in targeted regions. Eventually, a synergistic support system is established by combining mechanical supports (bolts/cables) with grouting, this integration achieves active load resistance, thereby enabling the deformation control efficiency to exceed 70% (relative to the peak displacement observed in Stage 4).

#### Absolute horizontal displacement of ribs and roof/floor

**Fig. 9:**
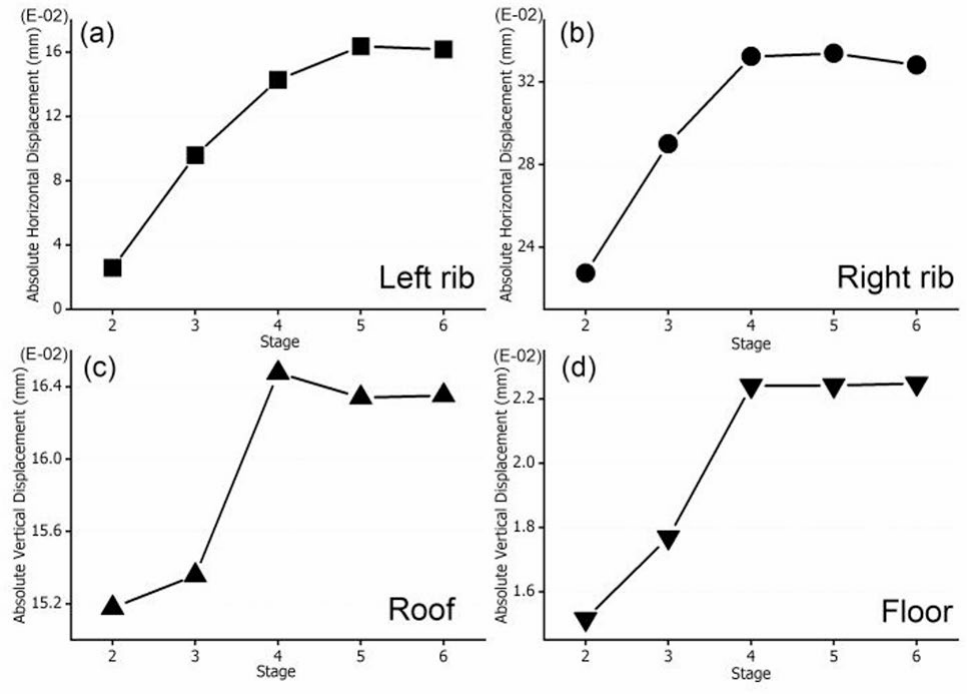
Absolute horizontal displacement of ribs and absolute vertical displacement of roof/floor. (**a**) AHD of left rib, (**b**) AHD of right rib, (**c**) AHD of roof, and (**d**) AHD of floor.

Figure 9 plots the absolute horizontal displacement (AHD) of ribs and absolute vertical displacement of roof/floor. In Fig. 9a, the displacement increases gradually from stage 2 to stage 5, then plateaus at stage 6. Stage 6 (rib bolts/cables installation) restrains further movement, it demonstrates the efficacy of mechanical supports.

In Fig. 9b, the displacement is significantly higher than the left rib (Fig. 9a), with a steep increase through stages 2–4, then stabilization in stages 5–6. Direct widening in stage 2 makes the right rib the primary deformation zone. Stages 2–4: Widening (Stage 2) + floor excavation (Stage 4) amplify stress, driving displacement. Stages 5–6: Grouting (Stage 5) and rib bolts/cables (Stage 6) stiffen and restrain the right rib, though residual displacement remains due to initial widening.

In Fig. 9c, The displacement is small until stage 4, where it spikes, then stabilizes. stage 4 (floor excavation) increases the roadway height, redistributing vertical stress and causing significant roof sag. Roof supports (Stage 3) are less effective against this height-induced stress, so displacement stabilizes but remains elevated.

In Fig. 9d, it gives the AVD of floor (floor heave), displacement increases steadily, with a jump at stage 4, then stabilizes. Stage 4 (floor excavation) removes support, triggering floor heave. Stages 5–6 (lower rib grouting, rib supports) provide lateral restraint, limiting further heave but not reversing it.

Widening (stage 2) and heightening (stage 4) are the primary drivers of increased displacement, with the right rib and floor/roof being most vulnerable. By carrying out intervention, then grouting (stages 3, 5) effectively stiffens coal-rib interfaces, reducing rib displacement. Roof supports (stage 3) control initial roof sag but are less effective against height-induced stress. Rib bolts/cables (stage 6) provide active tensile resistance, it stabilizes both ribs and limits post-stage 4 displacement growth. Therefore, it highlights the need for tailored reinforcement (grouting, mechanical supports) that adapts to changes in excavation geometry (width, height).

#### Load distribution along rock bolts and cable bolts

**Fig. 10:**
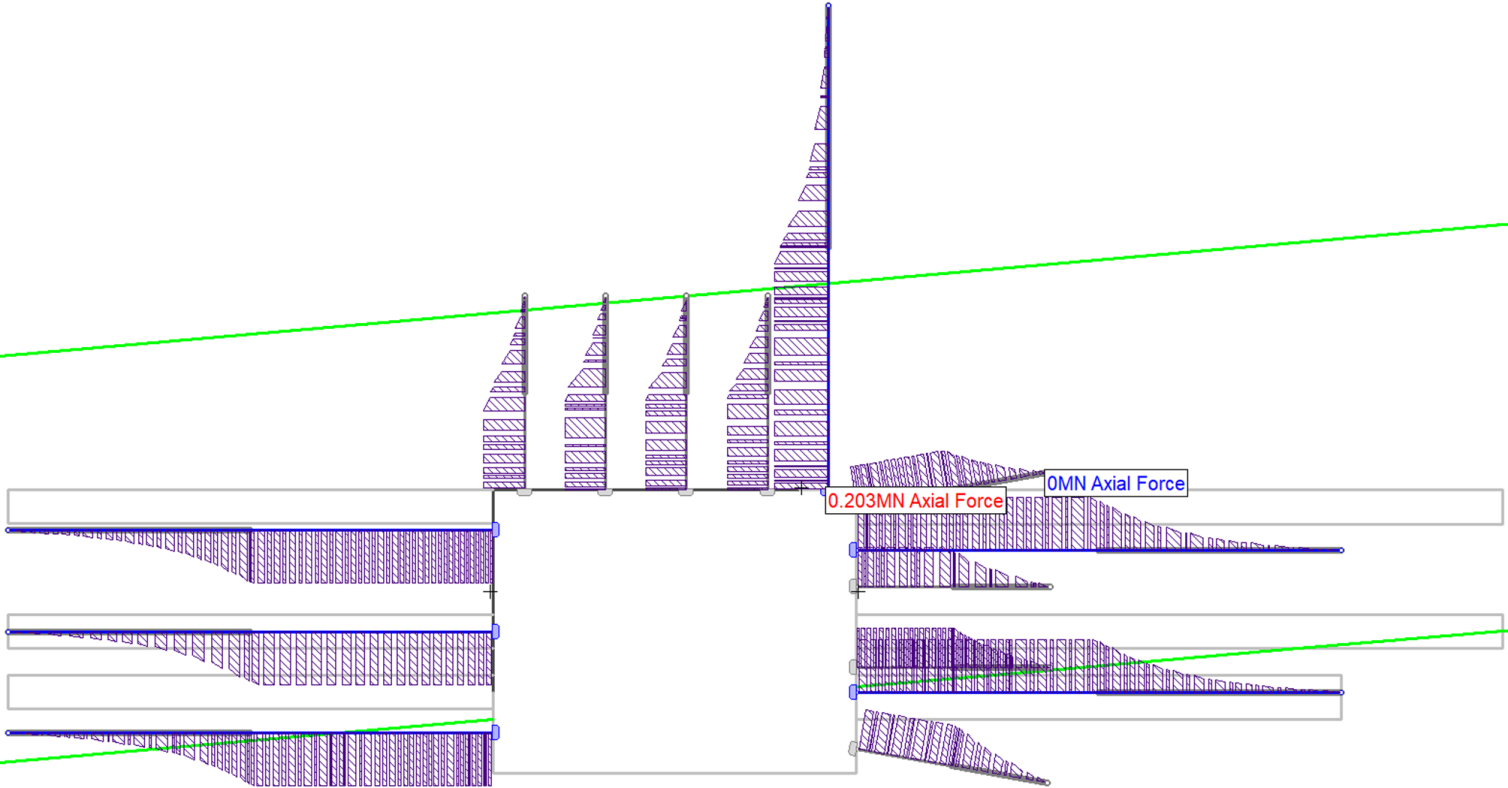
Load distribution along rock bolts and cable bolts.

Figure 10 shows the load distribution along rock bolts and cable bolts in stage 6, and the maximum loading point and the minimum loading point are also labelled (203 kN and 0 kN, respectively). For all bolts, axial forces taper off from the excavation face into the rock mass, this pattern shows that the bolt-rock bond is effectively transferring load from deforming rock to the bolts.

All bolts operate far below their tensile capacities, it indicates a reliable support system for long-term excavation stability. The right-side supports expresses higher forces, it reflects the asymmetric deformation from widening and floor excavation, and the model’s ability to capture geometry-driven stress patterns is thereby confirmed.

In addition, the maximum load (203 kN) for the top right cable bolt is relatively low if the loading capacity of cable bolt is considered. Therefore, it validates the stage-wise reinforcement strategy, grouting (stages 3, 5) first stiffens the rock mass, then bolts/cables (stage 6) provide active resistance without overloading.

### Numerical evaluation of supporting pattern in main ventilation roadway

**Fig. 11:**
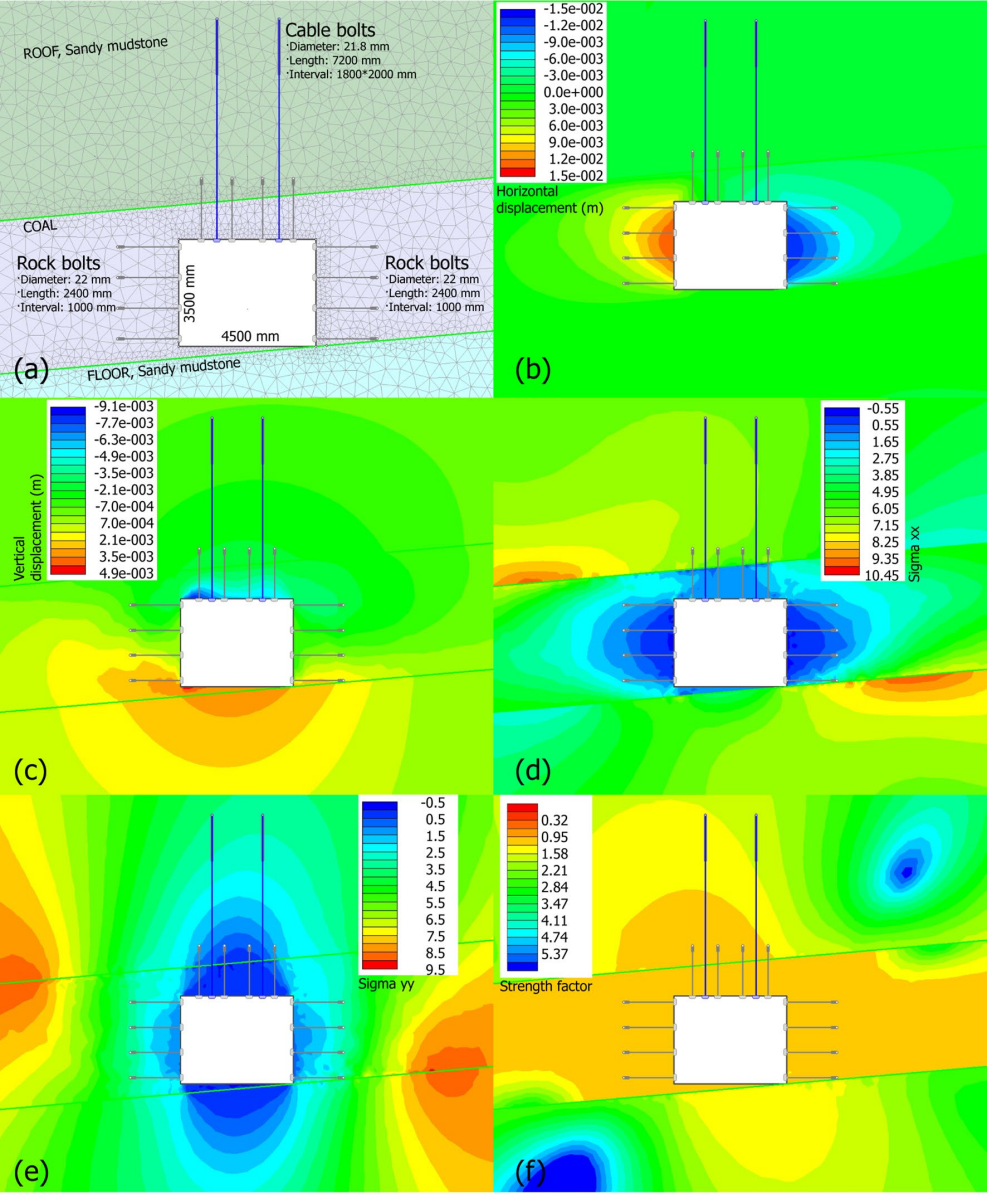
Original supporting pattern in main ventilation roadway and numerical results. (**a**) supporting pattern, (**b**-**c**) contour maps of horizontal displacement and vertical displacement, (**d**-**e**) contour maps of sigma xx and sigma yy, (**f**) contour map of strength factor.

The supporting pattern in the main ventilation roadway differs from that in the main haulage roadway, where a staged support strategy was adopted and analyzed in earlier sections. This section discusses the supporting pattern of the main ventilation roadway, with both the design and corresponding numerical results presented in Fig. 11. The model geometry is shown in Fig. 11a, the roadway is 4.5 m wide and 3.5 m high, the parameters for rib rock bolts: 22 mm diameter, 2400 mm length, spaced at 1000 mm intervals. The parameters for roof cable bolts: 21.8 mm diameter, 7200 mm length, anchored deep into the roof sandy mudstone.

For the horizontal displacement (Fig. 11b), the displacement ranges from − 15 mm to + 15 mm. Ribs exhibit symmetric convergence, with peak values localized near the excavation face. The 1 m spacing of rib rock bolts limits horizontal deformation, and it prevents coal spalling or rib collapse. The symmetric displacement confirms balanced load distribution across supports.

For the vertical displacement (Fig. 11c), roof sag (downward) reaches ~ 9 mm, and floor heave (upward) ~ 5 mm. Roof cable bolts (7200 mm length) anchor into the stiff roof sandy mudstone, so they effectively restrain large-scale roof sag. The vertical deformation is relatively moderate, and it validates the synergy between shallow rock bolts and deep cable bolts in controlling vertical movement.

For the σₓₓ (Fig. 11d), zones of stress relief (blue) near ribs indicate that rock bolts transfer horizontal loads from the coal seam to the surrounding rock mass. Peak stresses (red) are confined to distant, and it indicates a stable rock. The bolted rib zone acts as a “stress shield,” reducing stress concentration in the weak coal seam and preventing rib failure.

For the σ_γ__γ_ (Fig. 11e), the roof cable bolts transfer vertical loads from the excavation zone to the deep, stable roof sandy mudstone, and a stress-relief zones (blue) above the roadway is formed. This stress redistribution prevents roof bending and sag, leveraging the roof rock’s higher strength to support overburden loads.

For the Strength Factor (SF, Fig. 11f), the SF ranges from 0.32 to 5.37, with most zones showing SF ≥ 1. Only localized regions (e.g., very near the excavation face) have SF < 1. The support pattern (dense rib bolts + deep cable bolts) ensures the rock mass remains stable across the entire excavation. SF values above 1 confirm that failure is prevented, even in the weak coal seam. The elevated SF in the top-right and bottom-left regions is attributed to two key factors: (1) Top-right: The 7200 mm-long roof cable bolts anchor deep into the stiff sandy mudstone, they transfer overburden loads away from the weak coal seam and reducing stress concentration here, thus boosting SF. (2) Bottom-left: This area corresponds to the floor sandy mudstone and is indirectly restrained by the dense rib bolts, which limit lateral deformation and maintain the floor’s inherent stability, leading to higher SF.

Overall, the dense rib bolts limit horizontal convergence, while deep cable bolts restrain vertical movement. Bolts transfer loads from the weak coal seam to the stronger roof/floor sandy mudstone, avoiding stress concentration. In critical zones, the strength factor is above 1, so the design’s ability to resist failure is validated. It also demonstrates this multi-scale support strategy is a reliable solution for similar coal mine excavations.

### Numerical evaluation of supporting pattern in main conveyor belt roadway

#### Background and model establishment

**Fig. 12:**
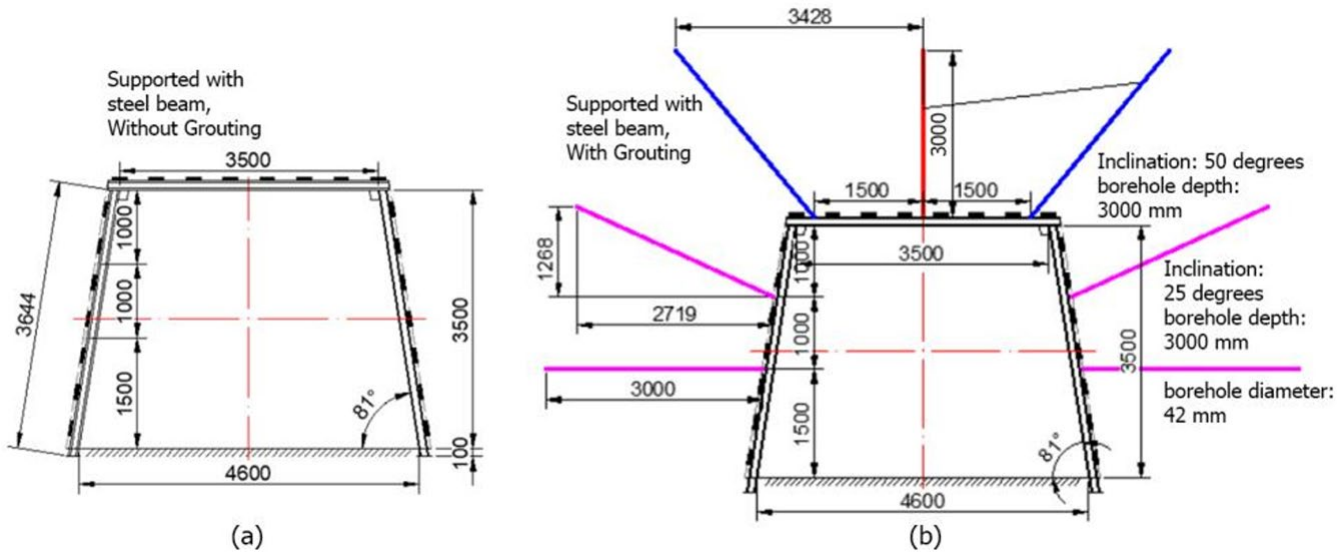
Support patterns for the highly fractured section: (**a**) supported with steel beams only, without grouting; (**b**) supported with steel beams combined with grouting.

For certain sections of the main conveyor belt roadway, the surrounding rock mass was severely fractured or highly disturbed. To address this condition, the cross-sectional geometry was modified to a trapezoidal shape, as shown in Fig. 12. Two support systems were implemented: steel beams alone (Fig. 12a) and a combination of steel beams with grouting (Fig. 12b). Initially, all sections were supported using steel beams only. Grouting was introduced progressively in response to observed deterioration of the surrounding rock; specifically, it was applied when the rock mass became highly fractured or unstable. The detailed geometric dimensions of the roadway and the grouting parameters are provided in Fig. 12.

In the simulation, the stratigraphic conditions were identical to those in the previous scenarios, with the only differences being the roadway geometry and support configurations. The simulation was conducted in three stages: the first stage involved excavation without support; the second stage included excavation with support implemented using the ‘liner’ function in Phase 2, with parameters defined according to the typical mechanical properties of mine-use steel beams; the third stage involved the installation of grouting, which was modeled in the same manner as in previous simulations. A grouting zone diameter five times that of the grouting borehole (42 mm) was assumed, resulting in a reinforced cylindrical column with a diameter of 210 mm. Therefore, all subsequent analyses are based on the differences observed across these three stages.

#### Absolute horizontal and vertical displacement

**Fig. 13:**
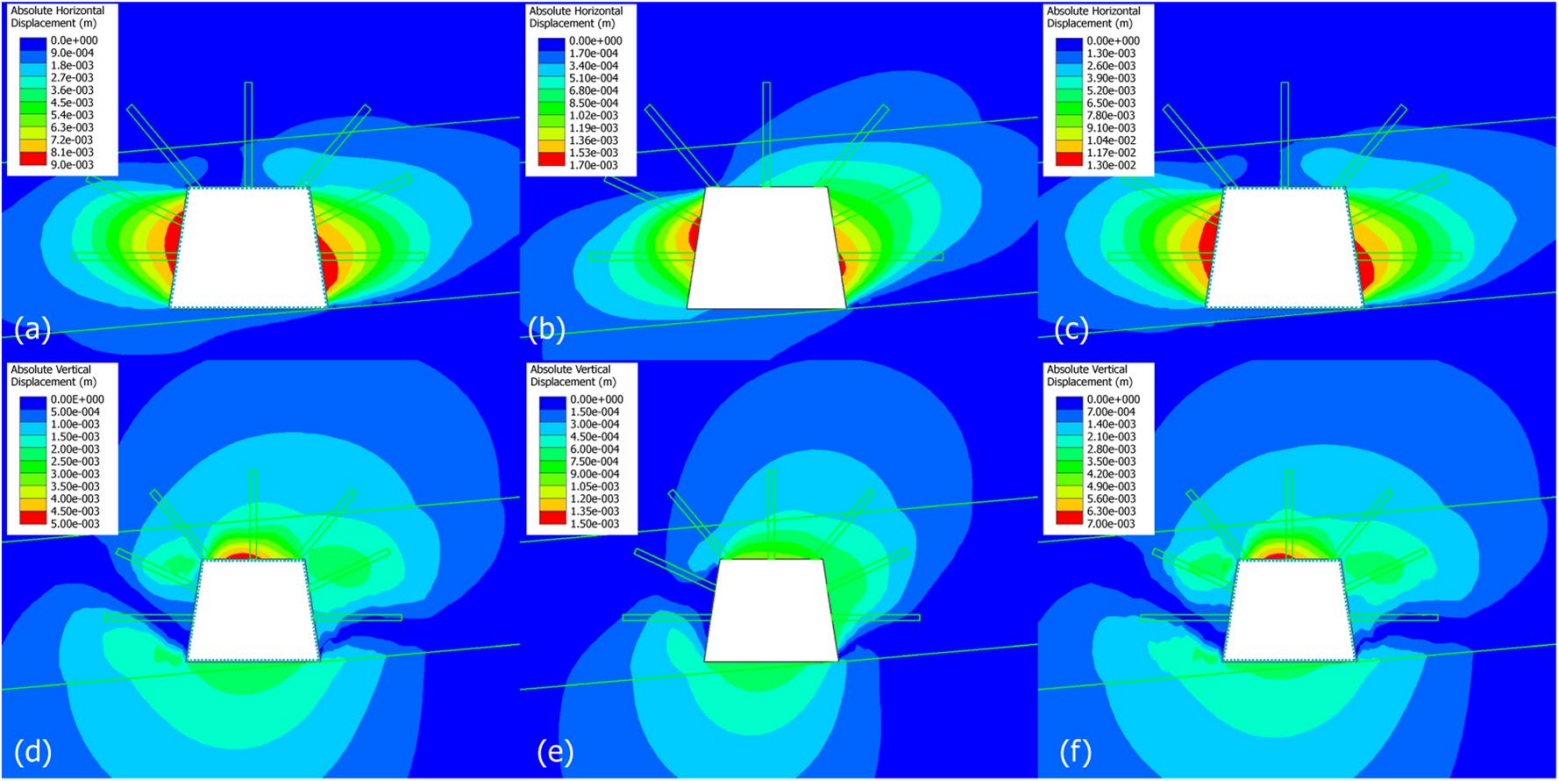
Contour maps of absolute horizontal and vertical displacement in three stages, (**a**-**c**) maps of horizontal displacement in stages 1–3, respectively, (**d**-**f**) maps of vertical displacement in stages 1–3, respectively.

Figure 13 presents the contour maps of absolute horizontal and vertical displacement in three stages. For horizontal displacement (Fig. 13a–c), the stage 1 is unsupported trapezoid, the peak horizontal displacement reaches 9 mm, with intense convergence at both ribs. The trapezoid’s wider lower rib (4.6 m) and narrower upper rib (3.5 m) create asymmetric stress, significant coal-rib movement can be noticed. The coal seam’s low strength cannot resist excavation-induced stress, leading to near-failure rib deformation. In stage 2, the roadway was by liner-supported, the displacement drops to 1.7 mm, it contributes a > 80% reduction from the previous scenario. The contour is uniformly light, indicating balanced load distribution. The “standard beam” liner acts as a rigid mechanical barrier, transferring rib loads to the surrounding rock and preventing coal spalling. After grouting in stage 3 (Fig. 13c), the displacement remains low (peak ~ 1.3 mm). Grouting (green bars) reinforces the rock mass around the liner, creating a “stiffened zone.” Grouting supplements the liner by anchoring into the coal and rock, therefore to restrict rib movement and enhance long-term stability.

Figure 13d-f showcases contour maps of vertical displacement. For the unsupported trapezoid (Fig. 13d), the peak vertical displacement reaches 5 mm, with roof sag and floor heave. The narrower roof (3.5 m) concentrates stress, leading to more pronounced sag than heave. Overburden stress drives deformation in the unsupported trapezoid, with the roof and floor failing to resist vertical loads. For liner-supported case (Fig. 13e), the displacement drops to 1.5 mm, more than 70% reduction compared with the previous case. The liner acts as a rigid structural beam, distributing vertical loads and preventing collapse. After the grouting (Fig. 13f), the displacement remains low (~ 2 mm), with grouting elements anchoring into the roof/floor sandy mudstone. This transfers vertical loads deeper into stable rock, further mitigating deformation.Field measurements from the trapezoidal roadway section at Daxi Coal Mine confirmed that floor heave was minimal, consistent with the simulated vertical displacement of approximately 2–5 mm. These on-site data validate the numerical results indicating negligible floor deformation. Therefore, the grouting enhances the liner’s performance by reinforcing the rock mass, and it creates a reliable system against long-term vertical stress.

Overall, the “standard beam” liner is critical for immediate stability, it reduces horizontal/vertical displacement by > 70%–80% compared to the unsupported stage. It acts as a rigid mechanical barrier, controlling both rib convergence and roof/floor movement. Grouting supplements the liner by stiffening the surrounding rock mass and improving load transfer, it also guarantees a synergistic supporting effects.

#### Horizontal and vertical stress

**Fig. 14:**
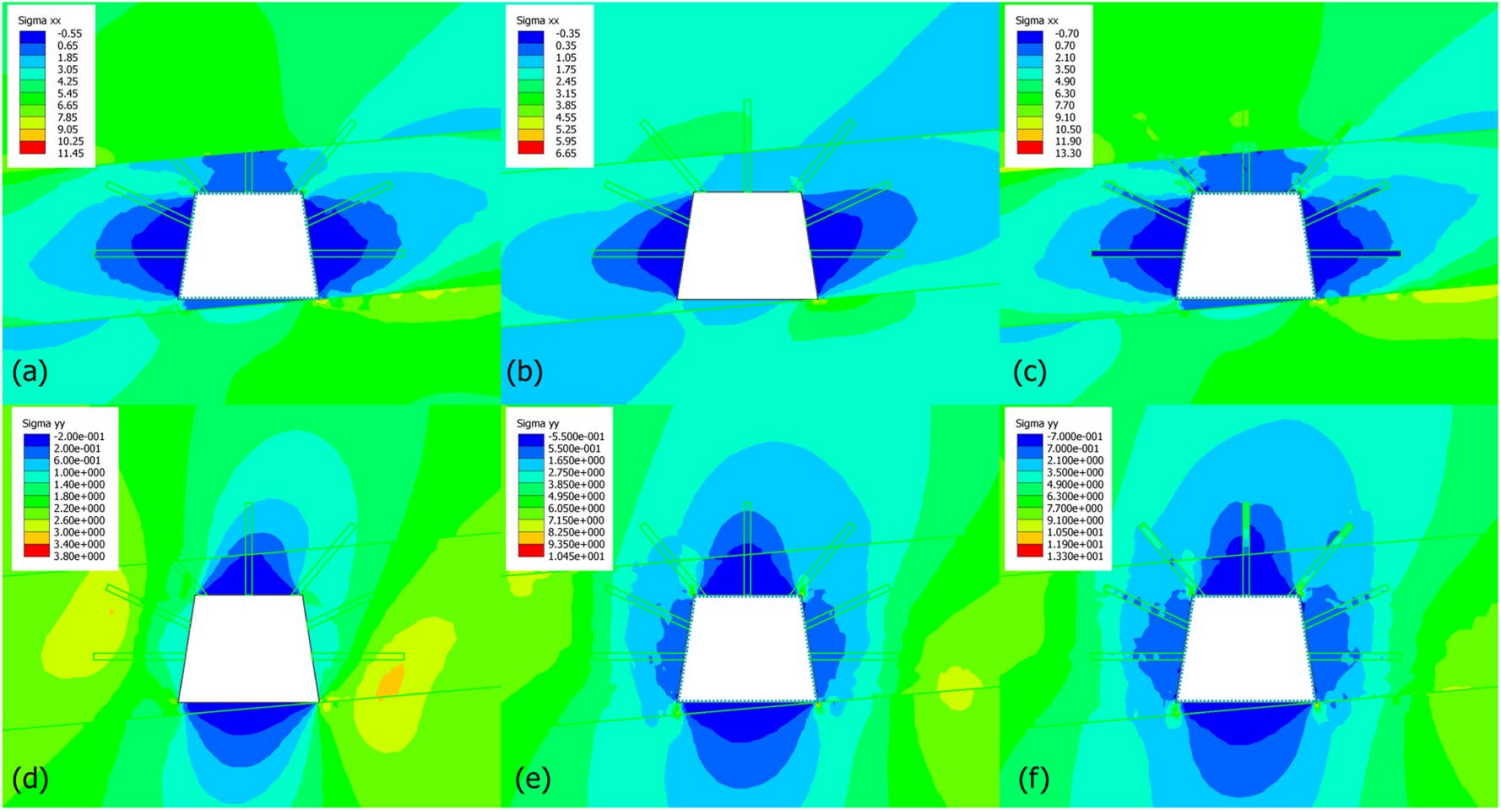
Contour maps of absolute horizontal and vertical stress in three stages, (**a**-**c**) maps of horizontal stress in stages 1–3, respectively, (**d**-**f**) maps of vertical stress in stages 1–3, respectively.

Figure 14 shows the contour maps of horizontal and vertical stress in three stages. For the horizontal stress (σₓₓ, Fig. 14a–c), extensive blue zones (stress relief) at coal ribs can be seen in the stage 1 (unsupported), as the weak coal seam compressive strength cannot resist excavation-induced horizontal stress. Peak stress (approximately 11.45 MPa) occurs in the sandy mudstone of the roof and floor. The trapezoidal shape intensifies stress relief in the coal mass, where the narrower upper rib and wider lower rib lead to asymmetric stress distribution. After liner-supported (Fig. 14b), the stress relief zones at ribs are reduced. The “Standard Beam” liner acts as a rigid mechanical barrier, transferring horizontal loads from coal to the liner and then to surrounding rock. Peak stress drops to approx 6.65 MPa because of load redistribution. The liner mitigates coal failure by intercepting horizontal stress, creating a more uniform stress field. After liner + grouting (Fig. 14c), the grouting elements (high-strength cement) stiffen the rock mass around the liner, further controlling stress distribution. Peak stress (approx 13.30 MPa) shifts to deeper rock, indicating stress is transferred more broadly into stable roof/floor sandy mudstone.

For vertical stress (σ_γ__γ_, Fig. 14d–f), significant blue zones (stress relief) at the roof and floor are seen in stage 1, as the coal seam’s low stiffness fails to support overburden loads. Peak stress (approx 3.80 MPa) is low, the coal’s ability to carry vertical stress is limited. The trapezoid’s narrower roof concentrates stress, but coal failure causes widespread stress relief. Liner-supported (Fig. 14e) in stage 2 indicates a reduction of stress relief at the roof/floor. The liner acts as a vertical load-bearing beam, distributing overburden loads to the ribs. Peak stress rises to approximate 10.45 MPa, showing the liner’s ability to transfer vertical loads to stronger rock. The liner prevents roof sag and floor heave by resisting vertical stress. In stage 3 (liner + grouting, Fig. 14f), grouting anchors the liner into the rock mass, enabling vertical stress to be transferred deeper into the roof/floor sandy mudstone. Peak stress increases to approx 13.30 MPa, indicating the combined system can mobilize the full strength of surrounding rock.

Therefore, The liner is critical for immediate stress redistribution, reducing both horizontal/vertical stress relief in coal by acting as a rigid mechanical support. Grouting stiffens the rock mass, allowing stress to be transferred deeper and more uniformly. This increases the system’s resilience, particularly in managing the trapezoid’s asymmetric stress patterns. The stress redistribution observed here directly correlates with reduced displacement (from prior analysis), therefore, the liner-grouting support effectively controls both stress and deformation in trapezoidal excavations.

#### Strength factor and yielded elements

**Fig. 15:**
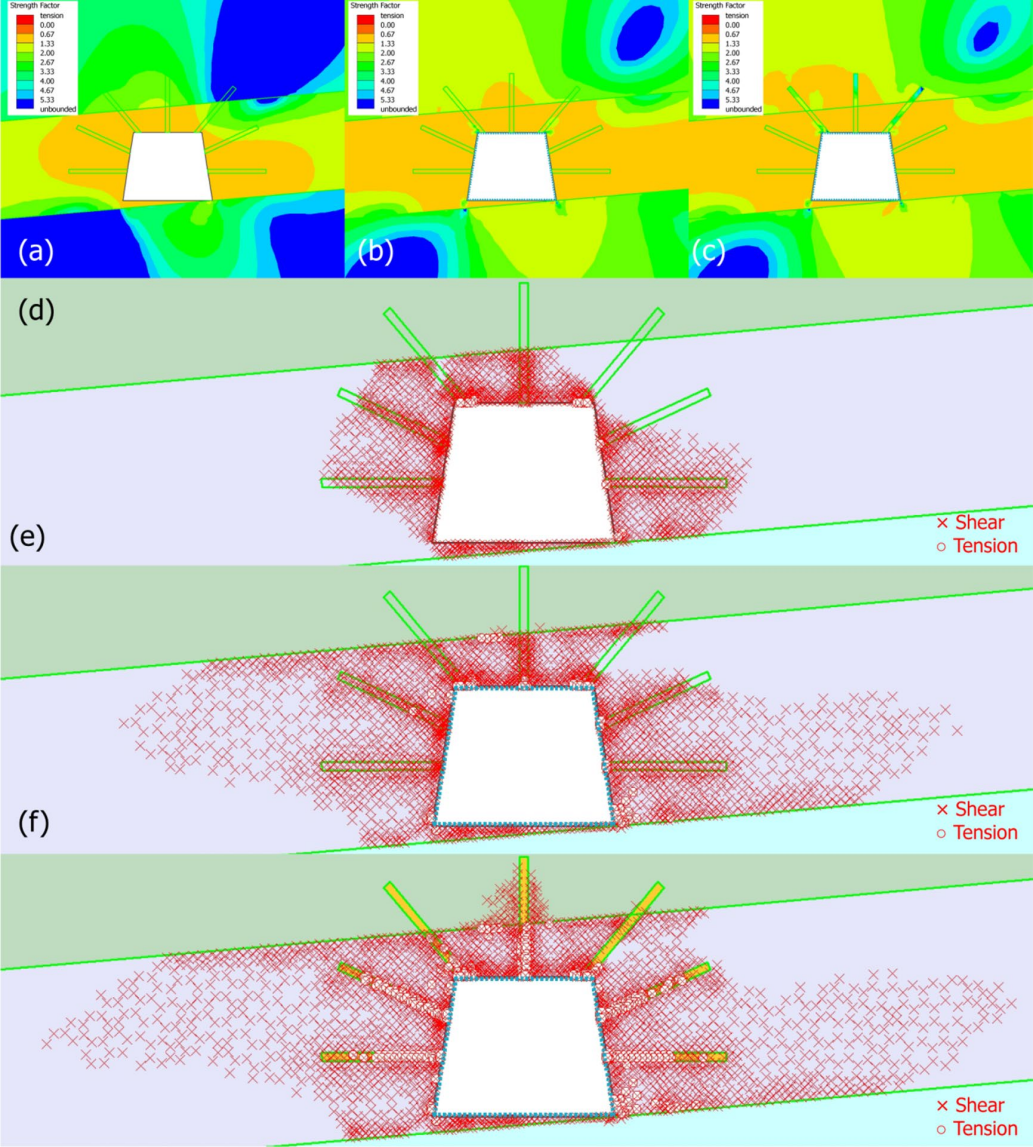
Contour maps of strength factor and indication of yielded elements in three stages. (**a**-**c**) maps of strength factor in stages 1–3, respectively, (**e**-**g**) maps of yielded elements in stages 1–3, respectively.

Figure 15 gives the contour maps of strength factor (SF) and indication of yielded elements in three stages. When the roadway is unsupported (Fig. 15a), SF is predominantly orange/red (SF < 1) around the roadway, especially in the coal seam. This reflects the coal’s low strength, which cannot resist excavation-induced stress, leading to widespread failure potential. When the roadway is liner-supported (Fig. 15b), SF improves significantly, with green/blue zones (SF1 ≥ 1) dominating. The “Standard Beam” liner acts as a rigid mechanical barrier, transferring loads from the coal to the liner and surrounding rock. As for liner + grouting (Fig. 15c), SF increases further, with extensive blue/green zones. Grouting (high-strength cement elements) stiffens the rock mass, distributing loads deeper into the roof/floor sandy mudstone, and enhancing overall stability.

For the yielded element, when the roadway is unsupported trapezoid (Fig. 15d), dense shear/tension failure zones surround the roadway, especially in the coal seam. The trapezoidal shape exacerbates stress concentration, leading to widespread failure. When the roadway is liner-supported (Fig. 15e), yielded zones are reduced but still present near the excavation. The liner restricts failure to a smaller area, demonstrating its role in controlling immediate deformation. In stage 3 (liner + grouting in Fig. 15f), yielded zones are drastically minimized, particularly around grouting elements. Grouting anchors into the rock mass, confining failure to very localized regions and synergizing with the liner to create a trustworthy support system.

The liner provides immediate mechanical support, reducing SF and yielded elements by acting as a load-transferring barrier. Grouting supplements the liner by stiffening the rock mass, further increasing SF and confining failure. This hybrid system leverages both mechanical (liner) and material (grouting) reinforcement. From stage 1 (widespread failure) to stage 3 (minimal failure), the support system demonstrates a clear ability to manage the trapezoid’s asymmetric stress patterns and ensure long-term stability. The results here demonstrate that a liner-grouting hybrid support is optimal for trapezoidal coal mine roadways, as it effectively mitigates failure and redistributes stress across multiple geological layers.

#### Liner axial force distribution

**Fig. 16:**
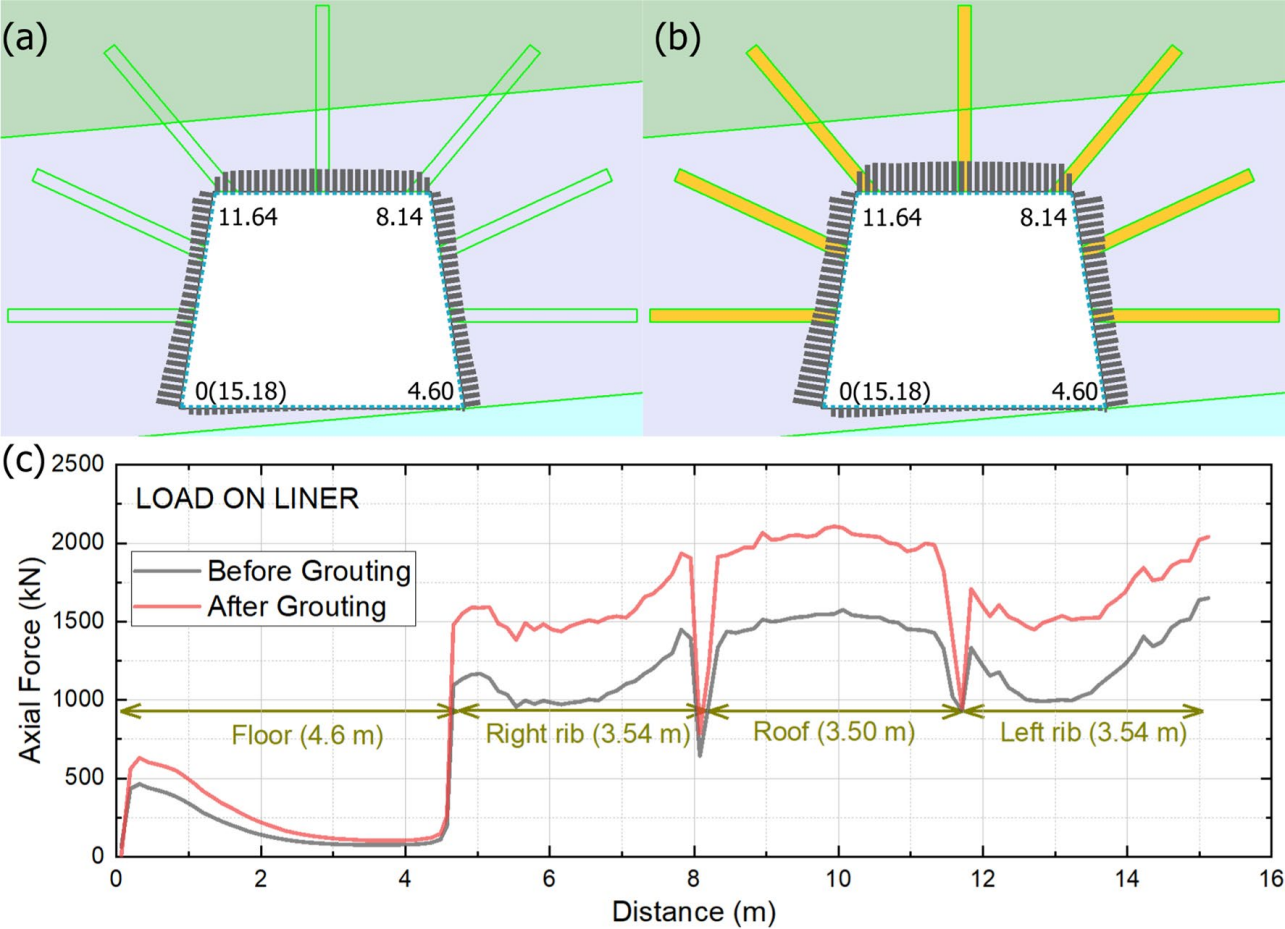
Liner axial force distribution, (**a**) overall force distribution before grouting, (**b**) overall force distribution after grouting (**c**) relationship of distance vs. axial force.

As previously mentioned and demonstrated in the earlier analysis, the liner-grouting hybrid support system is a reliable solution for supporting the fragmented section of the main conveyor belt roadway. This section discusses the loading characteristics of the liner before and after the grouting, with the mechanical results presented in Fig. 16.

Before grouting (Fig. 16a), the liner’s axial force (represented by gray bars) is relatively low. This reflects the weak coal seam’s inability to transfer significant load to the liner, as the coal’s low strength limit stress mobilization. After grouting (Fig. 16b), the axial forces increase substantially, even as the yellow grouting elements (high-strength cement) are introduced. Grouting stiffens the rock mass, enabling it to transfer more load to the liner. This demonstrates that grouting effectively “activates” the surrounding rock, turning it into a collaborative load-bearing component that amplifies stress transfer to the liner.

In Fig. 16c, the curve traces axial force around the trapezoid (floor → right rib → roof → left rib) before grouting (gray) and after grouting (red), the 0 m indicates the bottom left corner of the roadway. For floor (0–4.6 m), the axial force is lowest here, grouting has a minimal impact and the floor’s geometry inherently limits load intensity. For right rib (4.6–8.14 m, 3.54 m length), the axial force rises sharply, with the after grouting curve (red) peaking higher than before grouting (gray). The trapezoid’s inclined right rib concentrates stress, and grouting amplifies load transfer to the liner by stiffening this critical zone. For the roof (8.14–11.64 m, 3.5 m length), both curves peak here, with after grouting (red) reaching the highest force. The narrower roof (3.5 m) intensifies stress concentration, and grouting ensures this load is efficiently transferred to the liner, preventing roof sag. For the left rib (11.64–15.18 m, 3.54 m length), it basically mirrors the right rib, axial force is elevated, with grouting again enhancing load transfer. This symmetry confirms balanced support performance across both ribs.

In summary, grouting increases liner axial force by mobilizing the surrounding rock, and it transforms the support system from “liner-only” (dependent on a single component) to “liner-grouting” (a synergistic rock-liner-grout system). The yellow grouting elements stiffen the rock mass, and it transfers more stress to the liner. The trapezoidal roadway shape drives asymmetric loading, with the roof and ribs bearing higher forces. This aligns with principles of stress concentration in non-rectangular excavations.

## Conclusions

In this study, the roadway stability in Daxi Coal Mine’s weak rock conditions was investigated via field observations and numerical simulations. Roadway stability in weak coal seams requires stage-specific support and geometry-adapted hybrid schemes, such as grouting for rock stiffening, or bolts/cables for mechanical restraint. Based on the researches, following conclusions are established, and these findings provide a scientific and engineering reference for similar mining environments.

(1) Field investigations on the main haulage, ventilation, and conveyor roadways revealed severe instability, which included roof spalling, cracking, steel beam deformation, and roof-rib sagging due to the long-term abutment pressure from retreat mining. Drilling and borehole imaging confirmed the surrounding rock was highly fragmented, with no intact rock disks obtainable in key sections. These findings verified the inadequacy of initial support and the need for upgraded schemes to match the roadway’s service life with the mining panel.

(2) Numerical evaluation showed the original support (solely rock bolts) was insufficient, it could not control rib deformation, which was also a key driver of roof sag and irreversible roadway cross-sectional shrinkage. The scheme also failed to mitigate stress concentration in the weak coal seam, resulting in failure-prone zones and continuous expansion of shear-tension yield areas. This validated the necessity of multi-mechanism support integrating grouting and combined bolts/cables.

(3) A numerical model for the main haulage roadway was constructed to simulate six sequential stages: initial excavation, widening, primary support (roof bolts + rib grouting), heightening, supplementary grouting, and final rib reinforcement. The model captured dynamic stress and deformation evolution during progressive excavation, providing a reliable basis for quantifying support efficacy at each critical engineering step.

(4) The hybrid support pattern (rib rock bolts + roof cable bolts) for the main ventilation roadway achieved reliable stability. It restrained local coal spalling via dense rib bolts and transferred global overburden loads to stable deep rock via long roof cable bolts, eliminating stress relief zones at ribs and ensuring most regions remained stable. This confirmed the effectiveness of “local restraint + global load transfer” for rectangular roadways in weak rock.

(5) Given the highly fractured conditions in the main conveyor belt roadway, a trapezoidal roadway supported by the “steel liner + grouting” hybrid scheme was found to be effective. The steel liner initially reduced deformation by acting as a rigid barrier, while grouting further stiffened the fragmented rock, minimized yield zones, and shifted peak stress to deep stable rock. This scheme adapted well to the trapezoid’s asymmetric geometry, its suitability for highly fractured rock masses was validated.

## Data Availability

The datasets utilized and/or analyzed in the present study are available from the corresponding author upon reasonable request.
